# *A de novo* genome assembly of the dwarfing pear rootstock Zhongai 1

**DOI:** 10.1038/s41597-019-0291-3

**Published:** 2019-11-25

**Authors:** Chunqing Ou, Fei Wang, Jiahong Wang, Song Li, Yanjie Zhang, Ming Fang, Li Ma, Yanan Zhao, Shuling Jiang

**Affiliations:** 1 Key Laboratory of Horticultural Crops Germplasm Resources Utilization, Ministry of Agriculture, Research Institute of Pomology, Chinese Academy of Agricultural Sciences, Xingcheng, 125100 China; 2 Biomarker Technologies Corporation, Beijing, 101300 China

**Keywords:** Plant molecular biology, Plant breeding

## Abstract

‘Zhongai 1’ [(*Pyrus ussuriensis* × *communis*) × spp.] is an excellent pear dwarfing rootstock common in China. It is dwarf itself and has high dwarfing efficiency on most of main *Pyrus* cultivated species when used as inter-stock. Here we describe the draft genome sequences of ‘Zhongai 1’ which was assembled using PacBio long reads, Illumina short reads and Hi-C technology. We estimated the genome size is approximately 511.33 Mb by K-mer analysis and obtained a final genome of 510.59 Mb with a contig N50 size of 1.28 Mb. Next, 506.31 Mb (99.16%) of contigs were clustered into 17 chromosomes with a scaffold N50 size of 23.45 Mb. We further predicted 309.86 Mb (60.68%) of repetitive sequences and 43,120 protein-coding genes. The assembled genome will be a valuable resource and reference for future pear breeding, genetic improvement, and comparative genomics among related species. Moreover, it will help identify genes involved in dwarfism, early flowering, stress tolerance, and commercially desirable fruit characteristics.

## Abstract (summary)

**Table:** 

Measurement(s)	whole genome sequencing assay • transcription profiling assay • sequence_assembly • sequence annotation
Technology Type(s)	DNA sequencing • RNA sequencing assay • genome assembly • bioinformatics analysis
Sample Characteristic - Organism	Pyrus communis x Pyrus ussuriensis
Sample Characteristic - Environment	cultivated environment

Machine-accessible metadata file describing the reported data: 10.6084/m9.figshare.10029539

## Background & Summary

The pear (*Pyrus* spp.) is the third most abundantly cultivated fruit tree of temperate regions after the apple (*Malus pumila*) and grape (*Vitis vinifera*)^1,2^. There are at least 22 primary species of *Pyrus*, but only a few are widely cultivated for fruit production on a world scale, including *P*. *bretschneideri*, *P*. *communis*, *P*. *pyrifolia*, and *P*. *ussuriensis*^1^. The various *Pyrus* species differ widely in terms of growth and fruit characteristics. Based on their morphology and original distribution, the genus *Pyrus* can be divided into two major native groups, European pears (Occidental pears, *P*. *communis*) and Asian pears (Oriental pears, *P*. *pyrifolia*, *P*. *bretschneideri*, and *P*. *ussuriensis*)^3,4^.

Similarly to other fruit trees, the pear is characterized by a high degree of genetic heterozygosity and is mainly reproduced by grafting to maintain the fine properties of the cultivar. Therefore, the rootstock is very important in pear production and affects several aspects of plant resistance, growth, yield, and fruit quality^5–7^. The application of dwarfing rootstock, in particular, has been shown to reduce the length of the juvenility period and thus production costs; improve disease, insect, or virus resistance; and enhance fruit quality^8,9^. At present, quince rootstock is the most widely applied dwarfing rootstock for pears (*P*. *communis*) in Europe. However, quince rootstocks are suitable only for certain cultivars of *P*. *communis*, but cause incompatibility with scion and lime-induced chlorosis in most other cultivars^10–12^. Although other *Pyrus* dwarfing rootstocks have been bred, such as ‘OH × F’ series, ‘Fox’ series, ‘BP’ series, ‘Pyrodwarf’, and ‘Pyriam’ rootstocks^13–17^, a rootstock whose dwarfing efficiency is equivalent to that of the apple rootstock ‘M9’ and is suitable for most cultivated pear species (especially for *P*. *bretschneideri* and *P*. *ussuriensis*) has not been developed yet.

‘Zhongai’ series (NO. 1–NO. 5) dwarfing rootstocks have been bred by the Institute of Pomology, Chinese Academy of Agricultural Sciences (Xingcheng, in China) which are diploid and have the same number of chromosomes (2n = 34) with other diploid *Pyrus* species. These dwarfing rootstocks are all dwarf themselves, and can induce 50–70% dwarfing and early fruiting in the scions when used as inter-stocks. These rootstocks exhibit better resistance to cold and disease than most quince rootstocks, and have good compatibility with almost all cultivated European and Asian pear cultivars. They represent an excellent resource for the breeding of dwarfing rootstock and dwarf cultivars. Among them, ‘Zhongai 1’ has exhibited the best overall performance, with a dwarfing efficiency of about 65–70%; however, it is hard to root and can only be used as inter-stock. Importantly, its dwarfing mechanism remains unclear. To attain efficient breeding of new *Pyrus* rootstocks and dwarf cultivars, it is crucial to understand the molecular mechanism responsible for vigor control and precocity, a process that could be facilitated by assembling a high-quality genome for this rootstock. Even though two genome sequences have been assembled successfully in pears^1,18^, the trial materials originated from cultivars other than rootstock and belonged to different species. Additionally, they were based on next-generation sequencing technology limited to short reads (<400 bp). In this study, we combined third-generation sequencing technology (single-molecule sequencing), which produced long reads (average length of 8.74 Kb) with next-generation sequencing and Hi-C technologies, to assemble the *Pyrus* rootstock genome.

## Methods

### Plant material

**Fig. 1:**
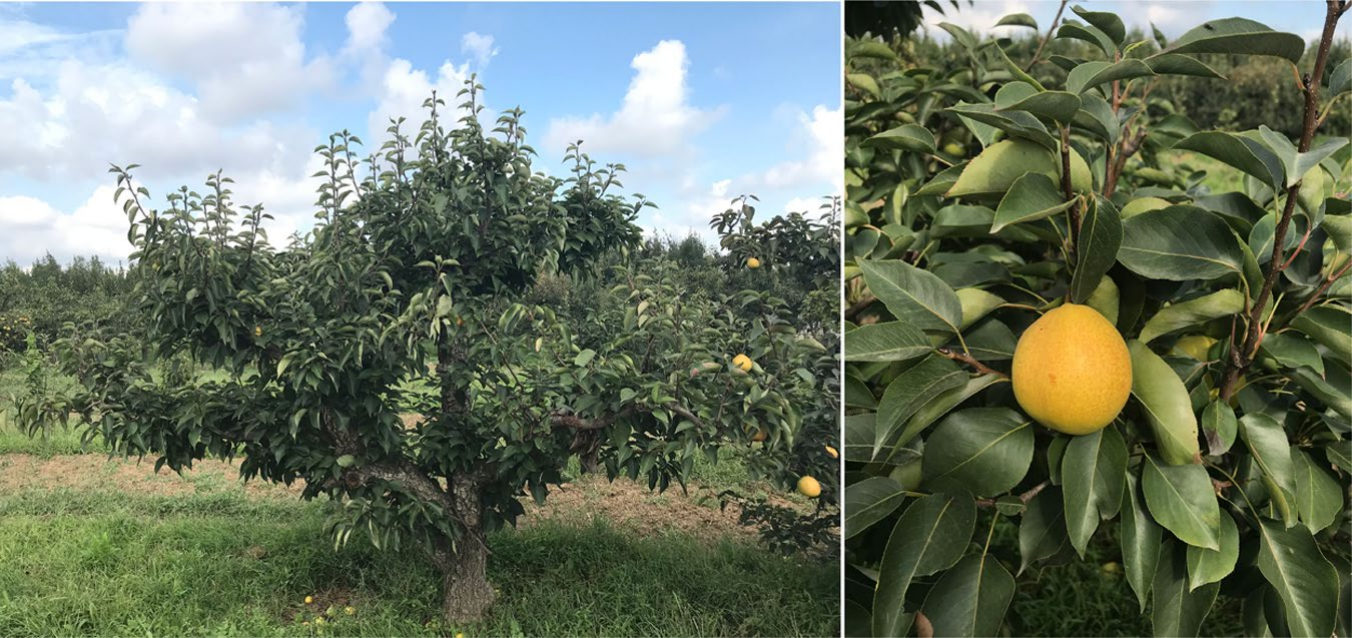
The Zhongai1’ pear tree and its fruit used in this study. Pictures were taken on September 11, 2018.

We used ‘Zhongai 1’ [(*P*. *ussuriensis* × *communis*) × spp.] as the trial material. ‘Zhongai 1’ is a naturally pollinated seedling of ‘Jinxiang’, which was selected from the cross of ‘Nanguoli’ (*P*. *ussuriensis*) × ‘Bartlett’ (*P*. *communis*). An individual grafted tree, whose rootstock is *Pyrus betulifolia*, grown in the orchard of the Institute of Pomology, Chinese Academy of Agricultural Sciences (120° 44′ 38′′E, 40° 37′ 9′′N) for over 15 years was selected (Fig. 1). All materials for sequencing, including leaves, shoots, flowers, and fruits, were collected from this tree.

### Estimation of genome size, heterozygosity, and repeat content

To estimate essential genome information, including genome size, heterozygosity, and repeat content, we collected the tender leaves from the selected ‘Zhongai 1’ tree, extracted genomic DNA using a modified CTAB method^19^, constructed a paired-end library of 270 bp according to the standard protocol provided by Illumina (USA), and sequenced it using the Illumina HiSeq X-Ten Sequencer (Illumina, USA). Paired-end reads had a length of 150 bp. After filtering and correction, 43.83 Gb of clean data were generated and are available at the Sequence Read Archive (SRA) of the National Center for Biotechnology Information (NCBI) under accession number SRR8382537. These data were used for genome size estimation, correction of genome assembly, and assembly evaluation, and were first analyzed by the kmer_freq_stat software (developed by Biomarker Technologies) with k-mer = 19. Based on the k-mer depth distribution (Supplementary Fig. S1), the highest peak was at a k-mer depth of 70, the genome size was estimated to be approximately 511.33 Mb, and the final cleaned data covered a genome depth of 85-fold. Repeat sequence content and heterozygosity rate were estimated to be 45.99% and 1.45%, respectively.

### PacBio SMRT sequencing

Genomic DNA was extracted from tender leaves using a modified CTAB method^19^ and sheared to about 20 Kb using a g-TUBE (Covaris, USA). Then, 20-Kb SMRTbell libraries were constructed using the SMRTbell Template Prep Kit 1.0 (Pacific Biosciences, USA) and the SMRTbell Damage Repair Kit (Pacific Biosciences), according to the manufacturer’ s instructions. Finally, the library DNA was sequenced at Biomarker Technologies Corporation (Beijing, China) on a PacBio Sequel sequencer (Pacific Biosciences) using P6-C4 sequencing chemistry (11 SMRT cells). After sequencing, Sequel raw bam files were converted into subreads in FASTA format using the standard PacBio SMRT software package. Low-quality and sequences shorter than 500 bp were filtered out, and a total of 7,224,701 PacBio subreads were obtained (NCBI SRA accession number: SRR8382538). That produced 63.16 Gb (123 × depth of the estimated genome) of single-molecule sequencing data with average reads length of 8,742 bp and max reads length of 102,449 bp (Supplementary Table S1).

### Genome assembly

The 63.16 Gb PacBio clean data were first assembled using Canu v1.5 software^20^ (corrected error rate = 0.045, cor out coverage = 70). A total of 4,977 contigs were generated with genome size of 987 Mb and a contig N50 of 0.42 Mb (Supplementary Table S2). The assembled genome was far larger than estimated. A second genome was assembled using WTDBG software (https://github.com/ruanjue/wtdbg) as follows: using the PacBio clean reads and the error-corrected reads from Canu, a draft and a better assembly were first generated with the command ‘wtdbg -i pbreads.fasta -t 64 -H -k 21 -S 1.02 -e 3 -o wtdbg’, after which the consensus assembly was obtained with the command ‘wtdbg-cns -t 64 -i wtdbg.ctg.lay -o wtdbg.ctg.lay.fa -k 15’. This generated 4,849 contigs with genome size of 603 Mb and a contig N50 of 0.24 Mb (Supplementary Table S3). A comparison of the two assembly results revealed that the second one was closer to the estimated genome size. To produce a more contiguous assembly, the two assemblies were merged using Quickmerge software^21^ (https://github.com/mahulchak/quickmerge) with the contigs from Canu as query input and those from WTDBG as ref input. The two contigs were aligned through Mummer v4.0.0 software^22^ (https://github.com/mummer4/mummer) with nucmer parameters ‘-b 500 -c 100 -l 200 -t 12’ and delta-filter parameters ‘-i 90 -r -q’. They were then merged through Quickmerge with parameters ‘-hco 5.0 -c 1.5 -l 100000 -ml 5000’. To obtain the final assembly, the draft assembly was polished twice. The first round of polishing adopted the quiver/arrow algorithm using the error-corrected PacBio single-molecule sequencing reads from Canu with 40 threads. The second polishing step adopted the Pilon algorithm v1.22^23^ (https://github.com/broadinstitute/pilon) using Illumina data with parameters ‘–mindepth 10 –changes–threads 4 –fix bases’. Finally, a genome of 510.51 Mb, composed of 1,207 contigs and with a contig N50 of 1.16 Mb, was obtained (Supplementary Table S4).

### Cluster, order, and orientation of pseudo-chromosomes by Hi-C

We constructed one Hi-C fragment library ranging from 300 to 700 bp using Nextera Mate Pair Library Prep Kit (Illumina, USA) according to the Reference Guide (15035209 v02) and sequenced the entries using the Illumina HiSeq X-Ten Sequencer to generate pseudo-chromosomes. We obtained 31.8 Gb clean Hi-C data (about 62 × depth of the estimated genome, NCBI SRA accession number: SRX5192481 and SRX5192482).

The mapped ratio of Hi-C reads to the assembled genome was assessed using BWA align 0.7.10-r789 (https://scicrunch.org/resolver/RRID:SCR_010910)^24^ with commands ‘bwa index -a bwtsw fasta, bwa aln -M 3 -O 11 -E 4 -t 2 fq1’ and ‘bwa aln -M 3 -O 11 -E 4 -t 2 fq2’. The result revealed that 78.45% of Hi-C reads (166,754,710) were mapped with the assembled genome, of which 44.47% (47,270,029) were uniquely mapped (Supplementary Table S5). Next, the 47.27 M unique mapped reads were analyzed using HiC-Pro 2.10.0^25^ with the command ‘HiC-Pro_2.10.0/scripts/mapped_2hic_fragments.py -v -S -s 100 -l 1000 -a -f -r -o’. The resulting number of valid interaction paired reads was 29,347,319, including 62.08% of unique mapped reads (Supplementary Table S6). This was sufficient for subsequent analysis.

Next, the final contigs were broken up to an equal length of 200 Kb, and were reassembled based on Hi-C data. Locations where contigs could not be reduced to the original assembled sequences, they were listed as candidate error areas. Locations of low Hi-C coverage depth in candidate error areas were identified as error locations and were corrected. The corrected genome sequences were preliminarily assembled using LACHESIS software^26^ with default parameters. The preliminary genome was further improved using PBjelly software^27^ with commands ‘PBSuite/15.2.20.beta/bin/Jelly.py’ and ‘PBSuite/15.2.20.beta/bin/fakeQuals.py’, and default parameters. A total of 512.78 Mb genomic sequences were obtained, with 1,198 contigs and a contig N50 of 1.39 Mb (Supplementary Table S7).

The contigs of the improved genome were then interrupted to an equal length of 200 Kb, and were reassembled and corrected again. The corrected sequences were assembled using LACHESIS software with the following parameters: (1) CLUSTER MIN RE SITES = 109; (2) CLUSTER MAX LINK DENSITY = 2; (3) CLUSTER NONINFORMATIVE RATIO = 2; (4) ORDER MIN N RES IN TRUN = 59; and (5) ORDER MIN N RES IN SHREDS = 57. Finally, sequences amounting to a total of 506.31 Mb (99.18% of the final contigs) were anchored onto the 17 pseudo-chromosomes; among these, the 475 ordered and oriented sequences corresponded to 427.18 Mb (83.54% of the total assembled genome) (Supplementary Table S8).

The quality of this final draft *Pyrus* genome was markedly improved compared to the last two versions from previous studies^1,18^. The final contig and scaffold number were 1,242 and 784, respectively, and the contig and scaffold N50 values were 1.28 Mb and 23.45 Mb, respectively (Table 1).

**Table 1:** Comparison of assembly results in three *Pyrus* species.

	(*P*. *ussuriensis* × *communis*) × spp. ‘Zhongai 1’	*P*. *bretschneideri* ‘Dangshangsu’^1^	*P*. *communis* ‘Bartlett’^18^
Assumed genome size (Mb)	511.33	527	600
Contig number	1,242	25,312	182,196
Contig length (Mb)	510.59	501.3	507.69
Max contig length (Mb)	6.53	0.3	0.13
Contig N50 (Kb)	1,277.34	35.7	6.57
Contig N90 (Kb)	202	—	—
Scaffold number	784	2,103	142,083
Scaffold length (Mb)	510.64	512.0	577.34
Max scaffold length (Mb)	31.94	4.1	1.29
Scaffold N50 (Mb)	23.45	0.54	0.09
Scaffold N90 (Mb)	0.44	—	—

### Repeat annotation

Because repetitive sequences are relatively poorly conserved among species, predicting a repetitive sequences for a particular species requires that a specific repetitive sequence database be constructed first. Therefore, we first built a repetitive sequence database for the ‘Zhongai 1’ pear using four types of software with default parameters: LTR FINDER v1.05^28^, MITE-Hunter^29^, RepeatScout v1.0.5^30^, and PILER v1.0^31^. All programs were based on the theory of structure prediction and *de novo* sequencing. Then, the database was classified using PASTEClassifier v1.0 software^32^ with default parameters and merged with the Repbase 19.06 (null) database^33^ as the final repetitive sequence database. Repetitive sequences amounting to a total of about 309.86 Mb (60.68% of the assembled genome) were predicted using RepeatMasker 4.0.5^34^ with the parameters ‘-nolow -no_is -norna -engine wublast -qq -frag 20000’ based on the prepared database of ‘Zhongai 1’ (Supplementary Table S9). Two types of repetitive sequences, Copia and Gypsy long terminal repeats, made up the largest proportion of the genome, corresponding to 20.49% and 23.18%, respectively.

### Gene prediction and functional annotation

Three methods were used here for predicting protein-coding genes in the assembled genome of the ‘Zhongai 1’ pear. 1) Prediction based on *ab initio* processing using Genscan^35^, Augustus v2.4^36^, GlimmerHMM v3.0.4^37^, GeneID 1.4^38^, and SNAP (v2006-07-28)^39^ software with default parameters and the *Arabidopsis* gene model as training model. 2) Prediction based on homologous species using GeMoMa v1.3.1 software^40^ with the protein databases of *P*. *bretschneideri* (GCF_000315295.1)^41^, *P*. *communis*^42^, *Malus* × *domestica* (GCF_000148765.1)^43^, and *Prunus persica* (GCF_000346465.2)^44^ from GenBank and the Genome Database for Rosaceae as references. 3) Prediction based on RNA sequencing using TransDecoder v2.0 (http://transdecoder.github.io), GeneMarkS-T v5.1^45^, and PASA v2.0.2^46^ software. The three predicted results were integrated using EVM v1.1.1 software^47^ with parameters ‘Mode: STANDARD, S-ratio: 1.13 score > 1000’ and the following weight values: PROTEIN OTHER 50, PROTEIN GeMoMa 50, TRANSCRIPT assembler-PASA 50, TRANSCRIPT Stringtie 20; ABINITIO PREDICTION Genscan 0.3, ABINITIO PREDICTION Augustus 0.3, ABINITIO PREDICTION GlimmerHMM 0.3, ABINITIO PREDICTION SNAP 0.3, ABINITIO_PREDICTION GeneID 0.3, and OTHER PREDICTION OTHER 100. Finally, a total of 43,120 genes were obtained, with an average length of 3,372 bp (Supplementary Tables S10–11).

The predicted genes were annotated against several functional databases by BLAST v2.2.31 (-evalue 1e^−5^), including NCBI non-redundant Nr and Nt databases (http://www.ncbi.nlm.nih.gov), KOG (ftp://ftp.ncbi.nih.gov/pub/COG/KOG), GO (https://www.uniprot.org/help/gene_ontology), KEGG (http://www.genome.jp/kegg), and TrEMBL (http://www.uniprot.org/). Results showed that 42,159 (97.77%) of all predicted genes could be annotated at least with one of the following databases: GO (44.03%), KEGG (30.02%), KOG (48.81%), TrEMBL (89.16%), Nr (92.81%), and Nt (96.94%) (Supplementary Table S12).

### Gene family and phylogenetic analysis

The protein sequences of the ‘Zhongai 1’ pear and other seven species of Rosaceae, including *P*. *bretschneideri*^41^, *P*. *communis*^42^, *Malus* × *domestica*^43^, *Prunus mume*^48^, *P*. *persica*^44^, *Prunus avium*^49^, and *Fragaria vesca*^50^ were clustered using OrthoMCL v2.0.9 software^51^ with parameters ‘Pep_length 10, Stop_coden 20, Percent Match Cut off 50, Evalue Exponent Cut off −5, Mcl 1.5 #1.2~4.0’. As a result, 39,270 genes of the predicted 43,120 genes of ‘Zhongai 1’ were clustered into 22,002 gene families, of which 291 were unique to ‘Zhongai 1’ (Supplementary Table S13 and Fig. S2).

To investigate the evolutionary relation of ‘Zhongai 1’ pear with the other above mentioned seven species of Rosaceae, 751 common single-copy genes from the seven species were used for phylogenetic reconstruction in PhyML^52^. HKY85 was chosen as the best model and was selected by the jmodeltest output with the command ‘java -jar, /share/nas2/genome/biosoft/jmodeltest/current/jModelTest.jar -d ./super_gene.phy -s 11 -i -g 4 -f -BIC -a -tr 8’ (Supplementary Fig. S3). Results showed that the test pear (named as *Pyrus*) had the closest genetic relationship with *P*. *bretschneideri*, *P*. *communis*, and *Malus* × *domestica* in that order of appearance, while *Fragaria vesca* was furthest away among the studied species. The estimated divergence time between this *Pyrus* test pear and *Malus* × *domestica* was estimated at 31.2 million years ago. Moreover, the *Pyrus* test pear included 2,657 expanded gene families and 453 contracted gene families (Supplementary Fig. S4).

### Collinearity analysis

**Fig. 2:**
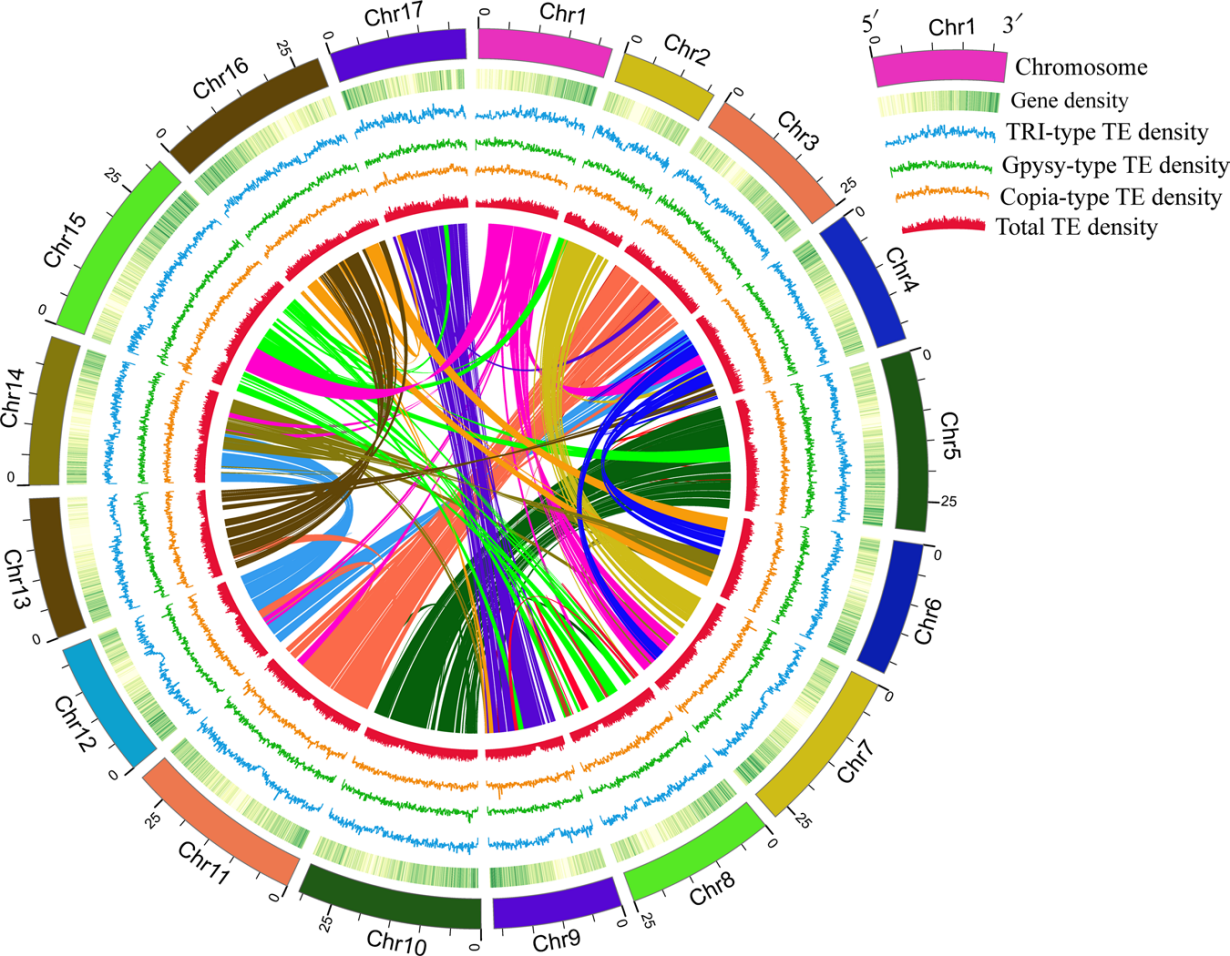
Synteny, gene, and transposable element (TE) distribution of the pear genome. As indicated in the inset, the rings indicate (from outside to inside) chromosomes (Chr), heat maps representing gene density (green), curve diagrams representing TRI-type TE density (blue), Gypsy-type TE density (green), Copia-type TE density (orange), and total TE density (red). Inside the figure, homologous regions of the pear genome are connected by colored lines representing syntenic regions identified by MCScan and mapped using Circos software. Seven data and one code files used to generate this figure are available at Figshare.

A previous study revealed that the pear shared a similar chromosome structure with the apple^1^. Collinearity analyses of chromosomes between ‘Zhongai 1’ pear and apple^53,54^ and between ‘Zhongai 1’ pear and ‘Dangshansuli’ pear (*P*. *bretschneideri*)^1,41^ were performed using MCScan software^55^. Results showed that all 17 pseudo-chromosomes of ‘Zhongai 1’ pear displayed good homology with the corresponding chromosomes of the apple and ‘Dangshansuli’ pear (Supplementary Fig. S5), so the naming order of the pseudo-chromosomes of ‘Zhongai 1’ was in line with that of the apple and ‘Dangshansuli’ pear. The genome of the latter two is characterized by good syntenic chromosome pairs^1,54^; similar syntenic chromosome pairs were found also in the genome of ‘Zhongai 1’ pear. They include Chr3 and Chr11, Chr5 and Chr10, Chr9 and Chr17, and Chr13, and Chr16 (Fig. 2), once again confirming the good assembly of our genome.

## Data Records

The Whole Genome Shotgun project has been deposited at DDBJ/ENA/GenBank under accession number SMOL00000000. The version described in this paper is version SMOL01000000^56^. Raw data from our genome project were deposited in the NCBI SRA database under Bioproject ID PRJNA494996^57^. The other files such as the contig order and arrangement in chromosome, assembled genome sequences, predicted CDS and protien sequences, repeat and gene annotation, all the data and code used to generate Fig. 2 are available at Figshare^58^.

## Technical Validation

To assure the quality of this assembled genome, it was assessed in terms of the following criteria before it was clustered by Hi-C. 1) Single-base error rate. A BLAST was performed using the corrected PacBio reads with the assembled genome, and inconsistent base numbers were counted. At the end, only 3,058 inconsistent bases were found, corresponding to 0.000599% of total contigs. 2) The integrity of core genes. A total of 458 conserved core genes of eukaryotes from the CEGMA 2.5 database^59^ were used to assess genome quality. Applying an identity of 70%, 442 of the 458 conserved core genes (96.50%) were found in our assembled genome, including 237 of the most conserved 248 genes (95.56%). 3) BLAST with PacBio subreads and Illumina clean reads. BLAST procedures were performed using the corrected PacBio subreads and Illumina clean reads with the assembled genome; the mapped ratios were 93.18% and 95.62%, respectively (Supplementary Table S14). 4) Benchmarking Universal Single-Copy Orthologs (BUSCO 2)^60^ assessment. Of the 1,440 single-copy orthologs conserved among all embryophytes, 1,284 (89.17%) complete BUSCOs were found in our assembly (Supplementary Table S15). All of the above steps ensured that our assembled genome possessed a relatively good integrity.

Hi-C technology enables the generation of genome-wide 3D proximity maps^26^ and has been successfully applied for constructing pseudo-chromosome sequences in many complex genome projects, including barely^61^, goat^62^, amaranth^63^, mosquito^64^, and peanut^65^. The assembly efficiency of Hi-C was very important for the quality of the draft genome. To assess assembly efficiency, the final assembled sequences were cut into equal lengths of 200-Kb bins, a thermography was made using the intensity of the interacting signal between any two bins (Supplementary Fig. S6). The intensity of the interacting signal was defined by the numbers of Hi-C read pairs covered in the bins. As the thermography shows, all the interacting signals were divided into 17 pseudo-chromosome groups. The intensity of the signal was stronger near the diagonal line, which was consistent with the Hi-C assembly theory and indicated that our draft genome was properly assembled.

High-density genetic linkage maps are also helpful in genome assembly. A previously published pear high-density genetic linkage map from the F1 population of ‘Red Clapp’s Favorite’ (*Pyrus communis* L.) × ‘Mansoo’ (*Pyrus pyrifolia* Nakai)^66^ was employed to assess the assembly quality of our genome. Small nuclear polymorphism markers on the genetic map were first divided into 3,122 blocks according to their genetic linkage relationship. Then, the physical location of the blocks in the pseudo-chromosome groups of our assembled genome was defined by the sequence information provided by the markers. Finally, Spearman correlation coefficients of the genetic and physical positions of the blocks in each pseudo-chromosome group were calculated. The results revealed very high consistency between our assembled genome and the map (Table 2, Supplementary Fig. S7), confirming the elevated reliability of our draft genome.

**Table 2:** Spearman correlation coefficients between the physical and genetic positions of the blocks in each pseudo-chromosome group.

Pseudo-chromosome groups	Spearman coefficient
1	0.9999973
2	0.9999981
3	0.9999984
4	0.9999983
5	0.9999999
6	0.9999985
7	0.9999994
8	0.9999992
9	0.9999991
10	0.9999978
11	0.9999991
12	0.9999980
13	0.9999932
14	0.9999989
15	0.9998377
16	0.9999993
17	0.9999982

To access the predicted result of gene, transcriptome data were aligned with genome sequences using TopHat v2.1.1 software^67^. Results revealed that 75.43% of transcriptome data were mapped to the exon region of genome sequences (Supplementary Table S16). This indicated that our prediction was well supported.

## Supplementary information

Supplementary Figures and Tables

## Data Availability

The public softwares used in this work, were cited in the Methods section. If no detail parameters were mentioned for a software, default parameters were applied with the guidance.
