# Supplementary material for: A de novo genome assembly of the dwarfing pear rootstock Zhongai 1
Source: Sci Data. 2019 Nov 25;6:281. doi: 10.1038/s41597-019-0291-3 (PMC6877535; doi:10.1038/s41597-019-0291-3)
Supplement: Supplementary file 1 — Supplementary Figures and Tables [file 41597_2019_291_MOESM1_ESM.pdf]

Content

Supplementary Figure S1. Distribution of 19-mer frequency in the sequencing reads....................2
Supplementary Figure S2. Ortholog clustering analysis of protein-coding genes in eight species of Rosaceae..3
Supplementary Figure S3. Phylogenetic tree of eight species of Rosaceae.....................................4
Supplementary Figure S4. Expansion and contraction of gene families in 'Zhongai 1' pear and other three species of Rosaceae...5
Supplementary Figure S5. Synteny of corresponding chromosomes between 'Zhongai 1' pear and 'Dangshansuli' pear, and 'Zhongai 1' pear and apple. Homologous regions of the corresponding chromosomes are connected by colored lines that represent syntenic regions identified by MCScan software...6
Supplementary Figure S6. Interaction frequency distribution of Hi-C links among chromosomes.. 7
Supplementary Figure S7. Collinearity map between the genetic linkage map and the assembled genome..8
Supplementary Table S1. Sequencing statistics from the PacBio platform......................................9
Supplementary Table S2. Summary statistics of the assembled genome by Canu software........... 9
Supplementary Table S3. Summary statistics of the assembled genome by WTDBG software........9
Supplementary Table S4. Summary statistics of the optimized genome...9
Supplementary Table S5. Mapped statistics of Hi-C read pairs with the optimized genome...........9
Supplementary Table S6. Summary statistics of different types of Hi-C read pairs........................10
Supplementary Table S7. Summary statistics of the improved genome by PBjelly software..........10
Supplementary Table S8. Assembly statistics of the final genome by LACHESIS software. The sequence length are the sum length of corresponding number contigs in which are without Ns. 11
Supplementary Table S9. Summary statistics of the repetitive sequences in the assembled genome..12
Supplementary Table S10. Summary statistics of gene prediction.. 13
Supplementary Table S11. Summary statistics of all predicted genes... 13
Supplementary Table S12. Summary statistics of gene annotations... 13
Supplementary Table S13. Summary statistics of the gene family cluster of eight species of Rosaceae..14
Supplementary Table S14. Mapped statistics of PacBio and Illumina reads with the optimized genome..14
Supplementary Table S15. Summary statistics derived from the BUSCO assessment of the assembled genome.. 14
Supplementary Table S16. Mapped statistics of transcriptome data with the final genome..........15

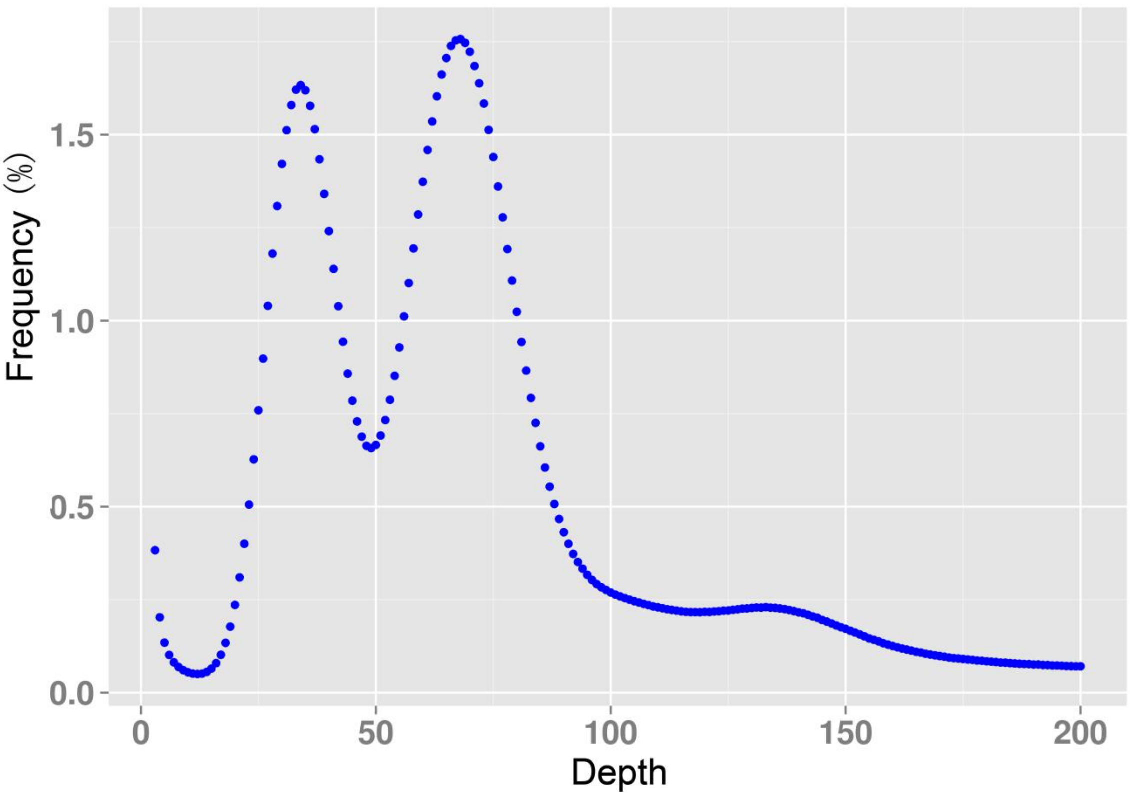

Supplementary Figure S1. Distribution of 19-mer frequency in the sequencing reads.

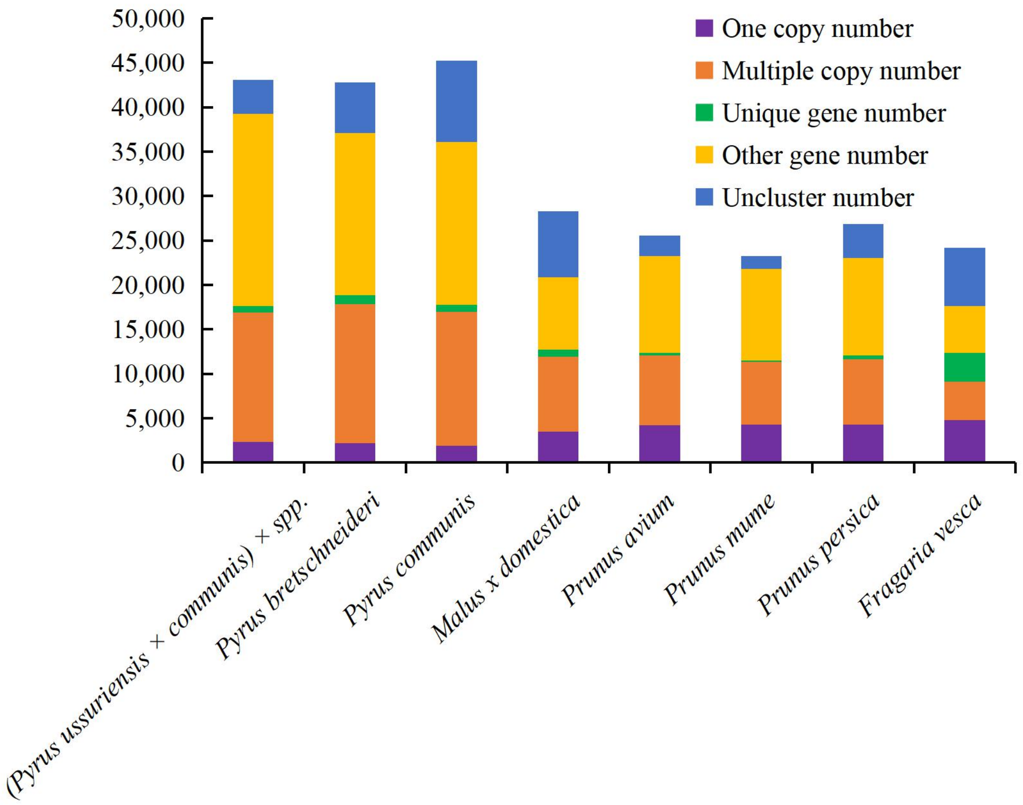

Supplementary Figure S2. Ortholog clustering analysis of protein-coding genes in eight species of Rosaceae.

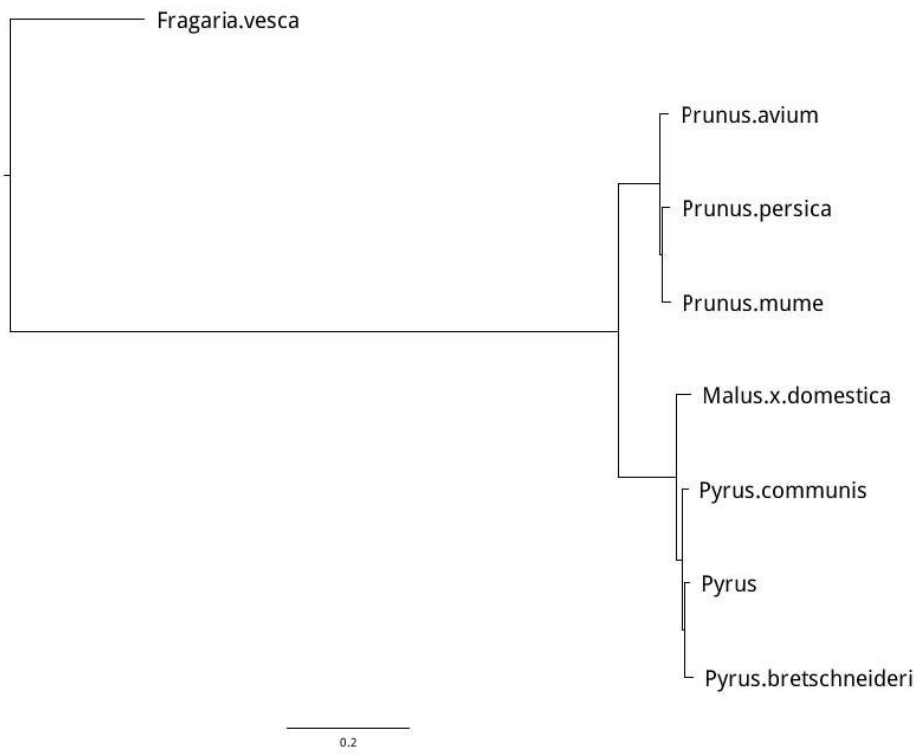

Supplementary Figure S3. Phylogenetic tree of eight species of Rosaceae.

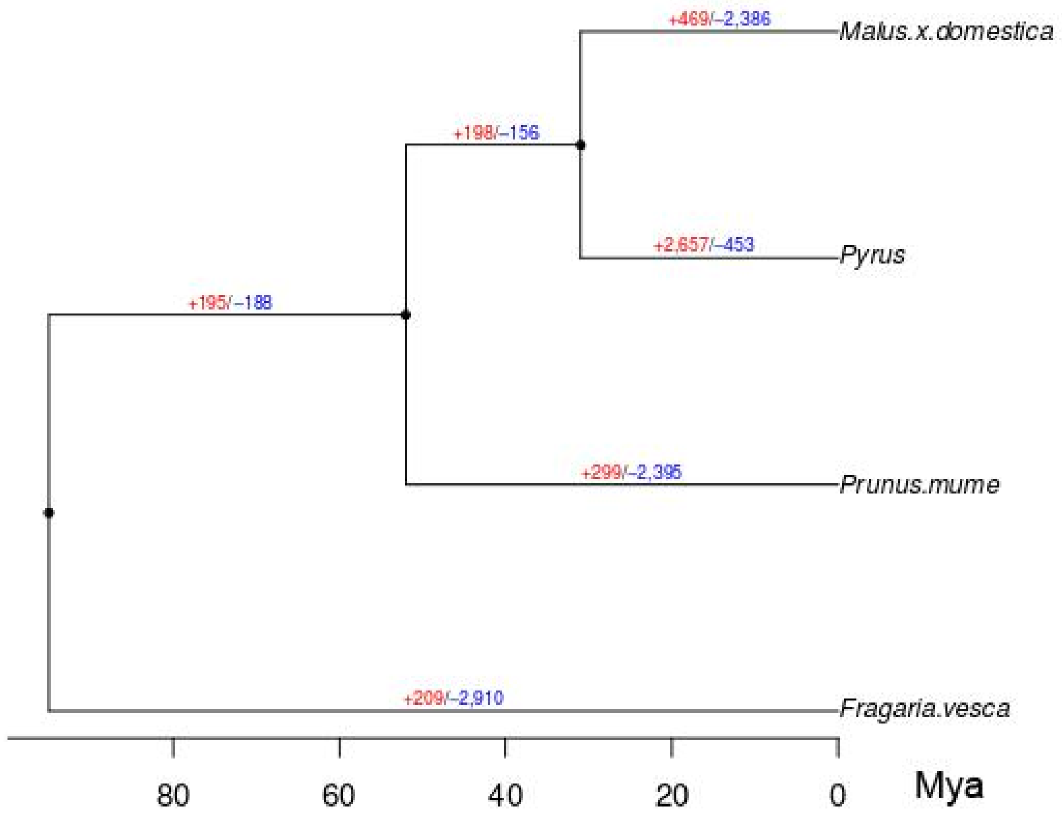

Supplementary Figure S4. Expansion and contraction of gene families in 'Zhongai 1' pear and other three species of Rosaceae.

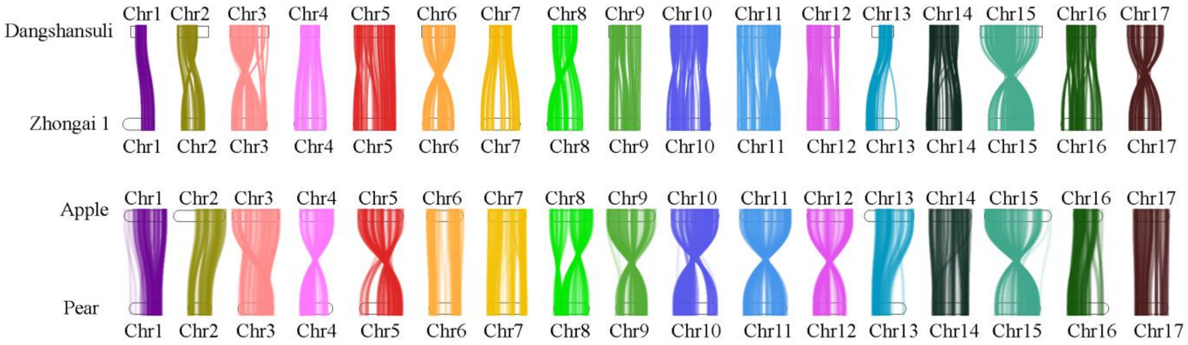

Supplementary Figure S5. Synteny of corresponding chromosomes between 'Zhongai 1' pear and 'Dangshansuli' pear, and 'Zhongai 1' pear and apple. Homologous regions of the corresponding chromosomes are connected by colored lines that represent syntenic regions identified by MCScan software.

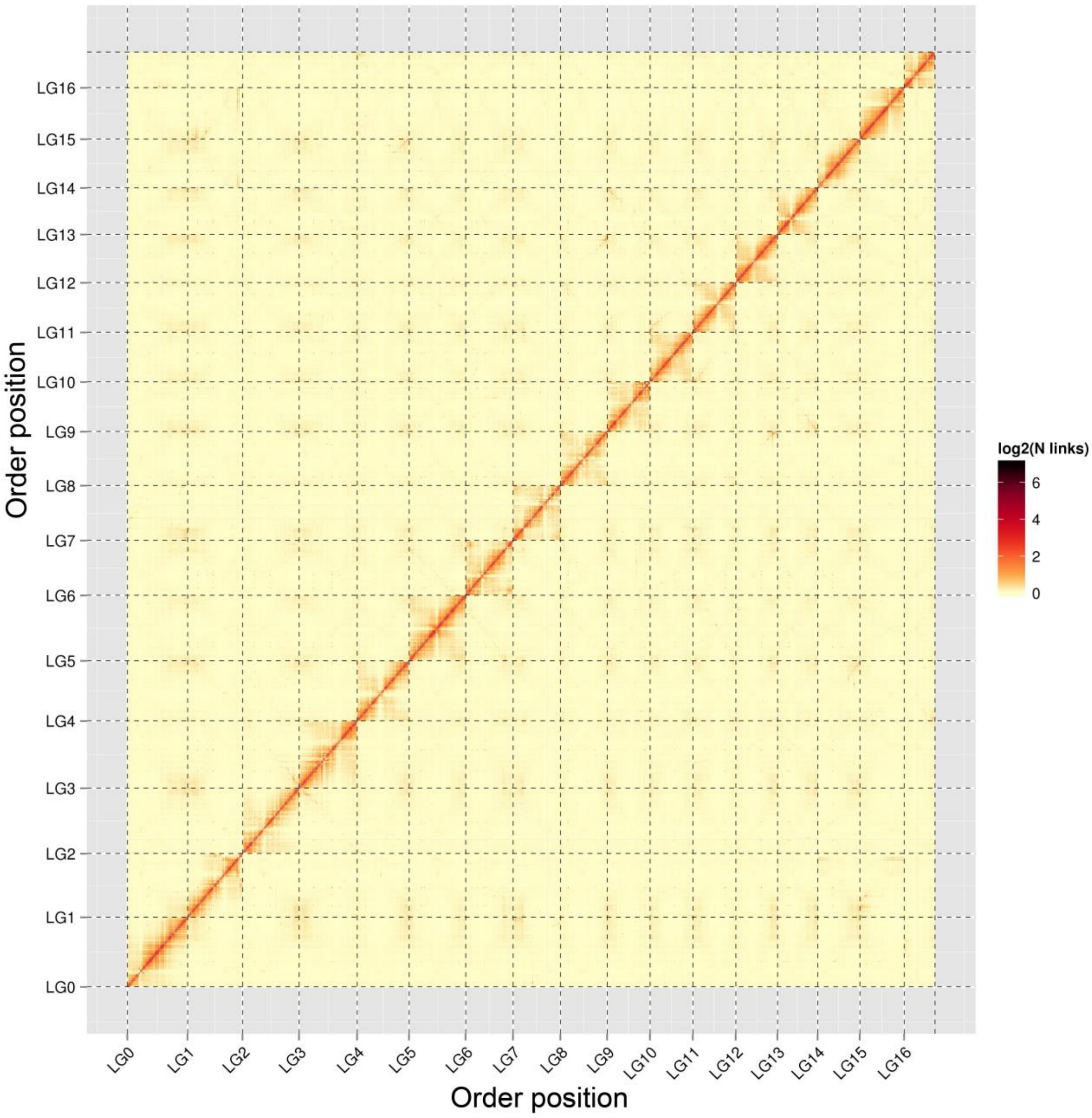

Supplementary Figure S6. Interaction frequency distribution of Hi-C links among chromosomes.

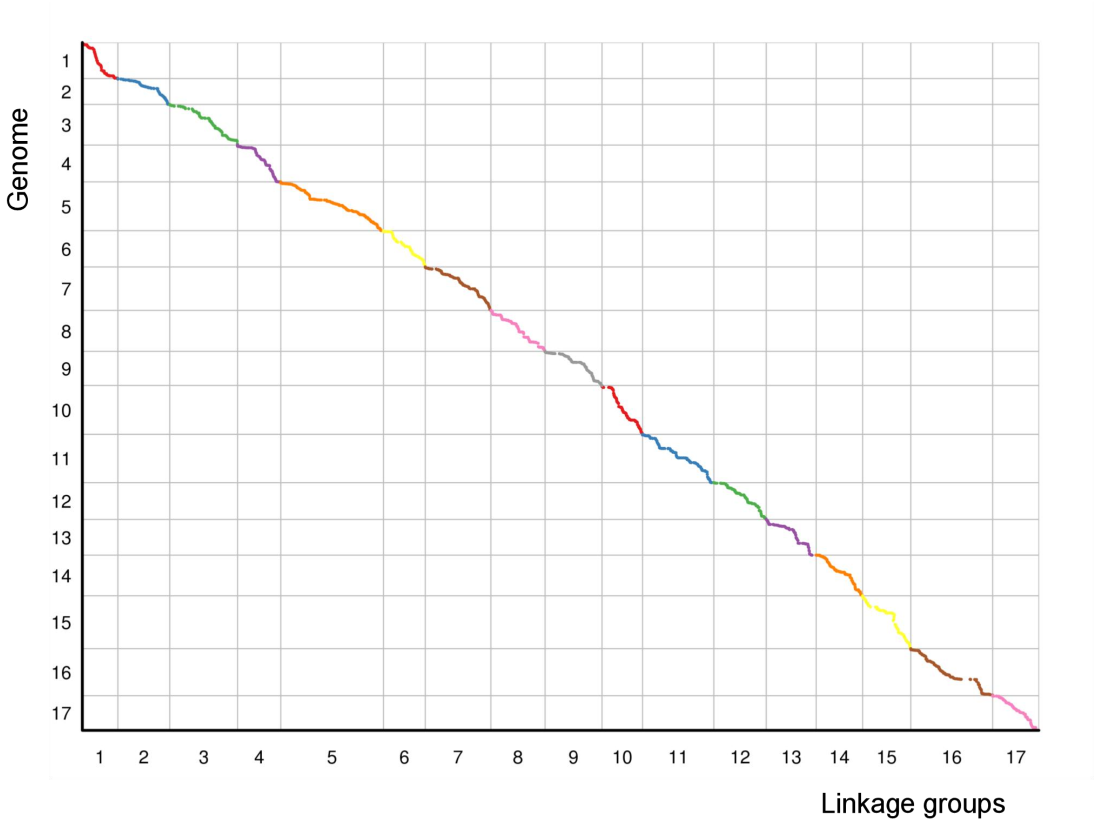

Supplementary Figure S7. Collinearity map between the genetic linkage map and the assembled genome.

Supplementary Table S1. Sequencing statistics from the PacBio platform.

Type	Reads number	Read bases (bp)	Mean reads length (bp)	Max reads length (bp)
Subreads	7,224,701	63,162,539,566	8,742	102,449

Supplementary Table S2. Summary statistics of the assembled genome by Canu software.

Contig number	Contig length (bp)	Contig N50 (bp)	Contig N90 (bp)	Contig max (bp)	GC content (%)
4,977	987,009,851	421,249	72,537	4,725,229	37.41

Supplementary Table S3. Summary statistics of the assembled genome by WTDBG software.

Contig number	Contig length (bp)	Contig N50 (bp)	Contig N90 (bp)	Contig max (bp)	GC content (%)
4,849	602,980,618	239,933	49,300	2,749,589	37.45

Supplementary Table S4. Summary statistics of the optimized genome.

Contig number	Contig length (bp)	Contig N50 (bp)	Contig N90 (bp)	Contig max (bp)	GC content (%)
1,207	510,505,314	1,156,855	242,305	6,540,539	37.38

Supplementary Table S5. Mapped statistics of Hi-C read pairs with the optimized genome.

Mapped type	Number	Ratio (%)
Total read pairs	106,286,983	100
Mapped read pairs	83,377,355	78.45
Unique mapped read pairs	47,270,029	44.47

Supplementary Table S6. Summary statistics of different types of Hi-C read pairs.

Type	Number	Ratio (%)
Unique paired alignments	47,270,029	100
Valid interaction pairs	29,347,319	62.08
Dangling end pairs	9,412,026	19.91
Re-ligation pairs	2,073,027	4.39
Self-cycle pairs	730,845	1.55
Dumped pairs	5,706,812	12.07

Supplementary Table S7. Summary statistics of the improved genome by PBjelly software.

Contig number	Contig length (bp)	Contig N50 (bp)	Contig N90 (bp)	Contig max (bp)	GC content (%)
1,198	512,777,937	1,394,002	214,472	6,534,904	37.39

Supplementary Table S8. Assembly statistics of the final genome by LACHESIS software. The sequence length are the sum length of corresponding number contigs in which are without Ns.

Pseudo-chromosome	Total clustered sequences			Ordered and oriented sequences		
	Number	Length (bp)	Gene number	Number	Length (bp)	Gene number
Chr1	64	24,457,932	2,041	32	22,288,274	1,846
Chr2	47	18,908,532	1,246	19	16,231,960	1,158
Chr3	61	30,937,624	2,723	25	24,998,000	2,227
Chr4	44	26,215,614	2,289	21	22,598,500	2,097
Chr5	85	36,913,704	3,254	33	29,909,632	2,668
Chr6	71	25,729,845	2,104	27	22,134,347	1,884
Chr7	74	34,074,470	2,815	32	27,576,311	2,378
Chr8	67	29,174,878	2,448	35	24,996,900	2,087
Chr9	80	25,332,901	2,263	33	21,378,220	1,944
Chr10	85	35,926,518	3,327	30	30,708,504	2,893
Chr11	58	32,612,592	2,812	28	29,880,766	2,553
Chr12	56	25,981,860	2,241	24	22,597,800	2,028
Chr13	34	25,184,980	1,627	19	23,449,246	1,443
Chr14	92	29,603,699	2,228	30	24,787,696	1,945
Chr15	60	36,970,598	3,760	30	31,936,364	2,887
Chr16	98	40,920,743	3,239	25	28,997,700	2,475
Chr17	77	27,360,698	2,366	32	22,706,679	1,972
Total of Chr1-17	1,153	506,307,188	42,783	475	427,176,899	36,485
Unclustered sequences	89	4,283,455	337	-	-	-
Total sequences	1242	510,590,643	43,120	-	-	-

Supplementary Table S9. Summary statistics of the repetitive sequences in the assembled genome.

Type	Number	Length (bp)	Percentage (%)
ClassI/DIRS	19,623	13,532,373	2.65
ClassI/LINE	47,363	14,315,243	2.8
ClassI/LTR	76,187	24,021,866	4.7
ClassI/LTR/Copia	158,166	104,609,945	20.49
ClassI/LTR/Gypsy	164,227	118,375,349	23.18
ClassI/PLE|LARD	73,475	16,505,716	3.23
ClassI/SINE	2,171	357,561	0.07
ClassI/TRIM	1,490	966,796	0.19
ClassI/Unknown	201	41,052	0.01
ClassII/Crypton	14	683	0
ClassII/Helitron	40,221	10,867,317	2.13
ClassII/MITE	5,421	1,166,213	0.23
ClassII/Maverick	5,246	1,481,224	0.29
ClassII/TIR	201,993	59,684,740	11.69
ClassII/Unknown	38,971	8,597,348	1.68
PotentialHostGene	7,751	1,822,969	0.36
SSR	531	89,217	0.02
Unknown	31,080	6,939,756	1.36
Total with overlap	874,131	383,375,368	75.08
Total without overlap	874,131	309,855,872	60.68

Supplementary Table S10. Summary statistics of gene prediction.

Method	Software	Species	Gene number
Ab initio	Genscan	-	14,160
	Augustus	-	31,898
	GlimmerHMM	-	46,352
	GeneID	-	28,028
	SNAP	-	38,402
Homology-based	GeMoMa	*Malus x domestica*	39,586
		Prunus persica	59,933
		Pyrus communis	129,911
		Pyrus bretschneideri	95,292
RNAseq	PASA	-	36,499
	GeneMarkS-T	-	36,168
	TransDecoder	-	51,943
Integration	EVM	-	43,120

Supplementary Table S11. Summary statistics of all predicted genes.

Soft ware	Gene number	Gene length (bp)	Average gene length (bp)	Exon length (bp)	Average exon length (bp)	Intron length (bp)	Average intron length (bp)
EVM	43,120	145,390,102	3,371.76	51,106,502	1,185.22	81,863,502	1,898.50

Supplementary Table S12. Summary statistics of gene annotations.

Database	Annotated number	Percentage (%)
GO annotation	18,985	44.03
KEGG annotation	12,943	30.02
KOG annotation	21,046	48.81
TrEMBL annotation	38,444	89.16
Nr annotation	40,020	92.81
Nt annotation	41,801	96.94
All annotated	42,159	97.77

Supplementary Table S13. Summary statistics of the gene family cluster of eight species of Rosaceae.

Species	Total gene	One copy number	Multiple copy number	Unique gene number	Other gene number	Cluster number	Uncluster number	Total family	Unique family
(*Pyrus ussuriensis* × *P. communis*) × *spp*	43,095	2,341	14,561	712	21,656	39,270	3,825	22,002	291
Pyrus bretschneideri	42,811	2,151	15,652	1,065	18,192	37,060	5,751	19,302	459
Pyrus communis	45,217	1,926	15,056	748	18,345	36,075	9,142	20,778	353
Malus x domestica	28,306	3,493	8,440	751	8,152	20,836	7,470	13,109	331
Prunus avium	25,541	4,170	7,870	290	10,896	23,226	2,315	15,120	121
Prunus mume	23,269	4,267	7,073	125	10,335	21,800	1,469	14,981	53
Prunus persica	26,873	4,275	7,334	438	11,002	23,049	3,824	15,841	182
Fragaria vesca	24,192	4,748	4,357	3,277	5,258	17,640	6,552	10,757	886

Supplementary Table S14. Mapped statistics of PacBio and Illumina reads with the optimized genome.

	PacBio reads	Illumina reads
Total number	3,346,775	299,449,134
Total length	33,438,820,422	-
Total mapped number	3,118,409 (93.18%)	286,335,182 (95.62%)
Total mapped length	30,674,324,877 (91.73%)	-
Proper mapped number	2,498,658 (74.66%)	255,415,570 (85.30)
Proper mapped length	23,343,900,847 (69.81%)	-

Supplementary Table S15. Summary statistics derived from the BUSCO assessment of the assembled genome.

	Number	Percentage（%）
Complete BUSCOs	1,284	89.17
Complete and single-copy BUSCOs	877	60.90
Complete and duplicated BUSCOs	407	28.26
Fragmented BUSCOs	20	1.39
Missing BUSCOs	136	9.44

Supplementary Table S16. Mapped statistics of transcriptome data with the final genome.

Type	Base number (bp)	Percentage (%)
Exon	5,699,197,428	75.43
Intron	1,232,530,411	16.31
Intergenic	623,765,598	8.26
Total	7,555,493,437	100
